# Ferroptosis: A New Promising Target for Ovarian Cancer Therapy

**DOI:** 10.7150/ijms.76480

**Published:** 2022-10-17

**Authors:** Hongjiang Zhao, Yuan Xu, Hongkai Shang

**Affiliations:** 1 The Fourth School of Clinical Medicine, Zhejiang Chinese Medical University, Hangzhou, China.; 2 Department of Gynecology, Affiliated Hangzhou First People's Hospital, Zhejiang University School of Medicine, Hangzhou, China.

**Keywords:** ferroptosis, ovarian cancer, target, therapy

## Abstract

Ferroptosis is a novel kind of regulated cell death distinct from autophagy, apoptosis, and necrosis; it is predominantly caused by the iron-dependent lipid peroxidation. According to studies, numerous conventional signaling pathways and biological processes are implicated in the process of ferroptosis. In recent years, researchers have shown that ferroptosis plays an important role in the genesis, development, and metastasis of malignancies, including ovarian cancer. Several studies have revealed that ferroptosis has synergistic effects with chemotherapy, radiotherapy, and immunotherapy in inhibiting the growth of ovarian cancer cells. This suggests that ferroptosis is important in ovarian cancer treatment and may be a new target. In this review, we summarize the features of ferroptosis, including its underlying basis and function in ovarian cancer, as well as its potential applications in the treatment of ovarian cancer.

## Introduction

Ovarian cancer (OC) is the primary cause of mortality from female reproductive cancers around the world 1. Moreover, because ovarian cancer is frequently diagnosed at an advanced stage, it presents a significant clinical challenge 2. Although overall survival at all stages of ovarian cancer has improved with advances in surgery, chemotherapy, and new immunotherapies, the 5-year overall survival rate for these patients remains at about 30% 3. Additionally, reliable biomarkers and therapeutic targets for the diagnosis of ovarian cancer are still lacking.

Ferroptosis is a recently identified form of regulated cell death driven by the iron-caused accumulation of lipid peroxide, and distinct from autophagy, apoptosis, and necrosis in morphology, biochemistry 4. Recent evidence demonstrates that ferroptosis modulates tumor genesis, development, and metastasis; thus, targeting ferroptosis may be a viable method for the treatment of ovarian cancer 5. In addition, several studies have revealed that ferroptosis has synergistic effects with chemotherapy, radiotherapy, and immunotherapy in inhibiting the growth of ovarian cancer cells. Chemotherapy is one of the standard treatments for ovarian cancer, which can effectively reduce the number of cancer cells and improve the prognosis of patients. However, some patients present with drug resistance or produce many side effects after treatment. At present, many studies have found that ferroptosis can synergize with chemotherapy to enhance the anticancer effect 6. Radiotherapy is a useful strategy for recurrent or refractory ovarian cancer. At present, many studies have found that ferroptosis can synergize with radiotherapy to enhance the anticancer effect 7. Immunotherapy's effectiveness in treating cancer has been widely recognized. However, immunotherapy is restricted since only one-third of ovarian cancer patients react to these agents. Recently, more than one kind of cell death mechanism has been shown to communicate with antitumor immunity. Wang et al. 8 found that the combination of ferroptosis induction and immunotherapy increased anticancer efficacy synergistically. Mechanistically, CD8+ T cells stimulated by immunotherapy induce ferroptosis in tumor cells. Ferroptotic cancer cells release high-mobility group box-1 (HMGB1) in an autophagy-dependent manner 9. As a significant damage-associated molecular pattern (DAMP), HMGB1 is an essential protein for the immunogenic cell death (ICD) of cancer cells 10. Ferroptotic cancer cells may trigger potent immune responses and enhance antitumor immunity through ICD. Therefore, ferroptosis plays an important role in the treatment of ovarian cancer. Further understanding of the related mechanism and clarifying the target of action are expected to provide a new method for the treatment of ovarian cancer.

In this review, we summarize the features of ferroptosis based on its underlying basis and function in ovarian cancer, as well as its potential applications in the treatment of ovarian cancer. Consequently, targeting ferroptosis might be a novel and promising method for eliminating ovarian cancer.

## The characteristics of ferroptosis

Morphologically, ferroptotic cells showed decreased mitochondrial volume, increased mitochondrial bilayer membrane density, decreased or absent mitochondrial cristae, and disruption of the outer mitochondrial membrane (OMM)^4^. Unlike necrosis, ferroptosis does not display swelling of the cytoplasm and organelles or breakdown of the plasma membrane. Unlike apoptosis, ferroptosis does not display agglutination of chromatin or the formation of apoptotic bodies. Unlike autophagy, ferroptosis does not display the formation of classical autophagosomes 11. In addition, microscopically recognizing ferroptotic cells is possible thanks to a unique phenotypic known as the ballooning phenotype, which is characterized by the development of a clear, spherical cell composed mostly of empty cytosol 12.

## The Mechanism of Ferroptosis

### Iron overload induces ferroptosis

Iron is usually found as ferrous (Fe^2+^) and ferric (Fe^3+^). Food iron primarily exists as Fe^3+^, which is insoluble and needs to be converted to Fe^2+^ for absorption. Fe^3+^ binds to transferrin (TF) in the serum and then is subsequently identified by the transferrin receptor (TFRC) in the cell membrane, which facilitates Fe^3+^ entrance into the endosome. The STEAP3 metalloreductase is an endosomal enzyme that reduces Fe^3+^ to Fe^2+^. Finally, Fe^2+^ is released from the endosomes to the cytosol and stored in the labile iron pool by solute carrier family 11 member 2 (SLC11A2/DMT1). Excess Fe^2+^ can be stored in the iron-storage protein ferritin, including ferritin light chain (FTL) and ferritin heavy chain 1 (FTH1), or excreted from the cell via the solute carrier family 40 member 1 (SLC40A1/ferroportin1/FPN) 13. In addition, excess iron can activate iron-containing enzymes (such as lipoxygenase) and produce reactive oxygen species (ROS) via the Fenton reaction, thereby promoting lipid peroxidation and ferroptosis 14. Therefore, intracellular iron plays a crucial role in the maintenance of cellular homeostasis.

### Lipid Peroxidation induces ferroptosis

Polyunsaturated fatty acids (PUFAs) are prone to lipid peroxidation and are necessary for ferroptosis. As one of the PUFAs, arachidonic acid or adrenic acid (AA/AdA) is the primary substrate of lipid peroxidation in ferroptosis 15. Acyl-CoA synthetase long chain family member 4 (ACSL4) and lysophosphatidylcholine acyltransferase 3 (LPCAT3) are essential in PUFA synthesis and remodeling 16. ACSL4 catalyzes the combination of free AA/AdA and CoA to create AA/AdA-CoA, while LPCAT3 stimulates the esterification of AA/AdA-CoA to membrane phosphatidylethanolamine (PE) to generate AA/AdA-PE. Different lipoxygenases can mediate lipid peroxidation, resulting in the hydroperoxides AA/AdA-PE-OOH and promoting ferroptosis 17. When the genes of ACSL4 or LPCAT3 are knocked out, lipid peroxidation decreases, inhibiting ferroptosis. Multiple membrane electron transport proteins, in particular, POR 18 and the NADPH oxidases (NOXs) 4, also contribute to ROS generation for lipid peroxidation in ferroptosis.

### Dysregulation of antioxidant defence induces ferroptosis

System Xc- is an amino acid antiporter formed by the functional subunit SLC7A11 and the regulatory subunit SLC3A2. It is an essential antioxidant system in cells and maintains the production of glutathione (GSH) by exchanging extracellular cystine with intracellular glutamate 4. Some small molecule compounds or drugs (such as erastin, sorafenib, and glutamate) can inhibit SLC7A11, leading to GSH depletion and triggering ferroptosis 19. In addition, some cells can promote the synthesis of GSH through the transsulfuration pathway (a pathway that bypasses the system Xc- and produces cysteine via methionine). Consequently, system Xc- inhibitors could not cause ferroptosis in these cells 20.

GSH is a necessary cofactor of the glutathione peroxidase 4(GPX4) antioxidant response and can cycle between oxidized (GSSG) and reduced (GSH) forms, allowing it to engage in redox biochemical reactions 21. Consequently, the targeted regulation of GSH is an essential mechanism of ferroptosis.

GPX4 can eliminate phospholipid peroxides and exerts a critical role in preventing the accumulation of ROS and maintaining lipid homeostasis, ultimately blocking ferroptosis 4. Consequently, the targeted regulation of GPX4 is an essential mechanism of ferroptosis.

AIFM2-CoQ10 22, GCH1-BH4 23 and ESCRT-III membrane repair systems 24 are non-GPx4 pathways and also play an important role in protecting against oxidative damage during ferroptosis. AIFM2 not only controls the generation of reduced CoQ10 but also activates the ESCRT-III membrane repair system 25.

## Ferroptosis in Ovarian Cancer-Associated Signaling Pathways

### MicroRNA (miRNA) induces ferroptosis

MiRNA is a non-coding RNA that plays a crucial role in posttranscriptional gene regulation 26. Emerging evidence indicates that alternations in miRNAs are identified to be related to various human cancers and regulation of miRNAs can render cancer cells vulnerable to ferroptosis 27.

Ma et al. 28 showed that *miR-424-5p* negatively regulates ferroptosis in ovarian cancer cells by targeting ACSL4. Downregulation of *miR-424-5p* increased ACSL4 expression, a positive mediator of ferroptosis, which sensitized ovarian cancer cells to erastin and RSL3-induced ferroptosis. Interestingly, they found that ACSL4 is remarkably overexpressed in ovarian cancer tissues and is positively correlated with the aggressive phenotypes of ovarian cancer. This may suggest that ACSL4 is required for maintaining the aggressive phenotypes of malignant cells in normal conditions, but it facilitates triggering ferroptosis following erastin treatment.

Cai et al. 29 found that long non-coding RNA (lncRNA) *ADAMTS9-AS1* prevents ferroptosis by targeting *miR-587*/SLC7A11 in the ovarian cancer cell line OVCAR3/CAOV-3. LncRNA is a gene expression regulator, involved in the progression of several cancers, including ovarian cancer 30. LncRNAs can attenuate miRNA-mediated inhibition of gene expression by sequestering miRNAs 31. In the study of Cai, inhibition of *ADAMTS9-AS1* promoted the expression of *miR-587*, and overexpression of *miR-587* enhanced the inhibition of SLC7A11 expression, ultimately promoted ferroptosis, and inhibited the proliferation and migration of ovarian cancer cells 29.

Lidocaine is widely used in clinics as a local anesthetic. However, recent studies have found that lidocaine can influence the development of a variety of tumors, including ovarian cancer 32. Some studies have even revealed that lidocaine can prevent tumor progression by regulating miRNA 33. Sun et al. 34 found that lidocaine induced ferroptosis in ovarian cancer cells by targeting *miR-382-5p*. Mechanistically, lidocaine upregulated *miR-382-5p*, which in turn suppressed SLC7A11 expression and triggered ferroptosis in ovarian cancer cells. Also, it was shown that lidocaine could stop SKOV-3 ovarian cancer cells from growing in mice. In tumor tissues, the expression of *miR-382-5p* was higher while the expression of SLC7A11 was lower.

### HIPPO induces ferroptosis

Hippo is an evolutionarily conserved pathway that can sense and regulate the density-dependent phenotypes of cancer cells. It converges into two transcriptional co-activators, YAP (Yes-associated protein 1) and TAZ (transcriptional coactivator with PDZ-binding motif) 35. Yang et al. 36 found that cell density in ovarian cancer cells controls how sensitive they are to ferroptosis. They then looked into how YAP and TAZ affect ferroptosis in ovarian cancer cells.

Yang et al. 36 found that *TAZ* regulates CAOV2 ovarian cancer cells' sensitivity to erastin-induced ferroptosis. *TAZ* knockdown confers ferroptosis resistance, while *TAZ* overexpression sensitizes cells to ferroptosis. Integrated genomic analysis revealed that *TAZ* regulates the target gene *ANGPTL4* directly to activate *NOX2* and sensitizes ferroptosis. It is known that NOX generates ROS (the hallmark of ferroptosis) 37. They verified that knockdown of *TAZ, ANGPTL4*, or *NOX2* reduced erastin-induced lipid peroxidation by measuring the lipid-based ROS. Thus, ferroptosis-inducing therapies may be especially effective against several TAZ-activated cancers.

A recent investigation has suggested that *YAP* plays a crucial role in regulating ferroptosis. Yang et al. 38 investigated the *YAP*-regulated ferroptosis in breast cancer cell line, renal cancer cell line, and lung cancer cell line. They found that overexpression of *YAP* makes cancer cells more susceptible to ferroptosis, whereas its knockdown gives ferroptosis resistance. Through integrated genomic approach, they identified that *YAP* regulates ferroptosis through regulating S-phase kinase-associated protein 2 (*SKP2*). Cells were protected from ferroptosis when *SKP*2 was repressed after *YAP* was knockdown. Therefore, the elevation of *YAP* expression can promote ferroptosis via *SKP2*. In addition, they also found that *SKP2* was repressed after *YAP* was knocked down in ovarian cancer cells. Whether the pathway of *YAP-SKP2*-ferroptosis exists in ovarian cancer has not been reported yet, and it needs to be further studied. We speculate that *YAP* can also promote ferroptosis via *SKP2* in ovarian cancer.

### Stearoyl CoA desaturase 1 (SCD1) induces ferroptosis

SCD1 is a rate-limiting enzyme that catalyzes the conversion of saturated fatty acids (SFAs) to monounsaturated fatty acids (MUFAs) and that is upregulated in numerous malignancies, including prostate, liver, and breast cancer 39. It has been shown that lipid metabolism is a crucial mediator of ferroptosis. Specifically, to trigger ferroptosis, increased amounts of phosphatidylethanolamine (PE) containing oxidized PUFAs are required 16. Given how important lipid peroxidation is to ferroptosis, regulating the lipid composition of cells is likely to perturb ferroptosis.

Tesfay et al. found 40 that SCD1 can protect ovarian cancer cells from ferroptosis. In the study, they found that SCD1 mRNA and protein was significantly increase in the genetic model of ovarian cancer stem cells, ovarian cancer cell lines, and tissue. Upon SCD1 inhibition, cells underwent both ferroptosis and apoptosis: inhibition of SCD1 decreased CoQ10(an endogenous membrane antioxidant that has been implicated in protection from ferroptosis) 41, While long-chain saturated ceramides increased and unsaturated fatty acyl chains in membrane phospholipids decreased, these changes are associated with apoptosis promotion. Since two death pathways are activated simultaneously, SCD1 inhibition may be an effective part of anti-tumor therapy. In addition, blocking SCD1 sensitizes ovarian cancer cells to ferroptosis inducers *in vitro and vivo* 40. Combining SCD1 inhibitors and ferroptosis inducers as a treatment for ovarian cancer is an area that needs more research.

Xuan et al 42 reported a novel cellular antioxidant system that SCD1/FADS2 could regulate GPX4 and the GSH/GSSG ratio to prevent excess ROS-mediated oxidative stress and ferroptosis. SCD1/FADS2 are the key iron-containing enzymes whose enzymatic activities are executed by binding to iron^43^. When SCD1/FADS2 is inhibited, its iron-binding capacity declines, leading to an accumulation of cellular iron. Experiments also revealed that inhibition of SCD1/FADS2 activities led to an increase in Fe^2+^ levels, which in turn resulted in ROS buildup and lipid peroxidation 42.

Kato et al. found 44 that MI-463(a Menin‑mixed‑lineage leukemia inhibitor) induces ovarian cancer cell line death through the induction of ferroptosis, which may be due at least in part to the inhibition of SCD1 activity. Liu et al 40 reported that Agrimonolide (the main bioactive polyphenol isolated from Agrimonia pilosa Ledeb) inhibits cancer progression and induces apoptosis and ferroptosis by targeting inhibition of SCD1 in A2780 and SKOV-3 ovarian cancer cell lines.

### p53 induces ferroptosis

*p53* regulates cell cycle checkpoints, DNA repair, and apoptosis as an important tumor suppressor 45. Previous research has confirmed that *p53* can promote ferroptosis by down-regulating SLC7A11 expression, thereby inhibiting the proliferation of lung cancer and breast cancer cell lines 46. However, there are few studies examining whether *p53* inhibits ovarian cancer cells by ferroptosis.

Hong et al. 47 found that PARP inhibitors promote lipid peroxidation and ferroptosis in ovarian cancer cells by downregulating SLC7A11 in a *p53*-dependent manner, thereby inhibiting tumor cancer cell growth.

Zhang et al. 48 found that SPIO-Serum (an iron-based nanomaterial) can induce lipid peroxidation and generate a large amount of ROS in ovarian cancer cells by downregulating GPX4 and system Xc-, ultimately leading to ferroptosis. Expression of *p53* can facilitate SPIO-serum-induced ferroptosis by inhibiting system Xc- to promote lipid peroxidation and ROS accumulation.

### SNAI2 induces ferroptosis

*SNAI2* plays a multifunctional role in cancer progression, including the induction of tumorigenesis, invasion, and metastasis 49. Previous studies have found that *SNAI2* is activated in ovarian cancer cells and has the potential to promote lymphovascular diffusion 50. However, the potential mechanism between ferroptosis and *SNAI2* in ovarian cancer remains unclear.

Jin et al. 51 observed elevated *SNAI2* expression in ovarian cancer cells, especially in SKOV3 cells. *SNAI2* knockdown significantly suppressed cell viability, migration, invasion, and accelerated cell apoptosis by inducing ferroptosis in SKOV3 cells. In addition, *SNAI2* knockdown significantly inhibited SLC7A11 protein expression. So, *SNAI2* knockdown may induce ferroptosis and inhibit the progression of ovarian cancer by downregulating SLC7A11.

### Nuclear factor erythroid 2-related factor 2 (Nrf2) induces ferroptosis

Overexpression of *Nrf2* has been linked to decreased response to anticancer treatments and a worse prognosis for patients 52. Recently, *Nrf2* has been shown to be an antioxidant transcription factor that protects tumor cells against ferroptosis 53. However, further research is required to determine whether ferroptosis is involved in *Nrf2*-induced resistance to anticancer therapies and a worse prognosis.

Liu et al. 54 found that *Nrf2* could activate the transsulfuration pathway by upregulating Cystathionine-β-synthase (CBS), a rate-limiting enzyme of the transsulfuration pathway 55, resulting in resistance of ovarian cancer cells to erastin-induced ferroptosis. Therefore, activation of *Nrf2*/CBS is a novel anti-ferroptosis mechanism. Inhibition of the transsulfuration pathway by down-regulating *Nrf2* can increase the sensitivity of ovarian cancer to ferroptosis.

Gai et al. 56 found that carboxymethylated pachyman (a carboxymethylated derivative of pachyman derived from Poria cocos) can induce ferroptosis by downregulating *Nrf2* mRNA, and reducing Nrf2, HO-1, GPX4 protein levels in ovarian cancer cells. Therefore, the *Nrf2* gene is expected to be a potential therapeutic target for ovarian cancer.

## Ferroptosis in Ovarian Cancer Therapy

### Chemotherapy and ferroptosis

When it comes to female gynecological cancers, ovarian cancer is by far the most lethal. Although chemotherapy can effectively reduce the number of cancer cells, residual tumors still persist 57. Importantly, residual tumors are accompanied by decreased apoptotic response, enhanced antioxidant defence, and enhanced efflux mechanisms, which result in the development of drug resistance 58, 59. Due to the shortcomings of chemotherapy, other regulatory death pathways, such as ferroptosis, have been investigated to improve cancer treatments. It has been shown that ferroptosis plays a crucial role in the chemotherapy of ovarian cancer and enhances the anticancer impact of cisplatin in the treatment of ovarian cancer.

Cheng et al. 6 showed that cisplatin triggered numerous forms of cell death in the ovarian cancer cell, including ferroptosis. Erastin elevated ROS levels and induced ferroptosis to enhance the cytotoxicity of cisplatin. Combination treatment with cisplatin and erastin appears to increase therapeutic results while reducing adverse effects *in vitro and vivo* models of ovarian cancer.

Wang et al. 60 observed that the expression of *Frizzled 7 (FZD7)* was elevated in platinum-tolerant ovarian cancer cells. The overexpression of *FZD7* could decrease the sensitivity to platinum. Mechanistically, *FZD7* overexpression activated the oncogenic factor *Tp63*, driving the upregulation of GPX4 and protecting cancer cells from chemotherapy-induced oxidative stress. Interestingly, inhibition of GPX4 caused these platinum-tolerant (Pt-T) ovarian cancer cells to become more sensitive to platinum and undergo ferroptosis. This suggests that targeting *FZD* is a novel treatment for platinum-tolerant cancer cells.

Multidrug resistance (MDR) is connected with overexpression of drug efflux transport ATP-binding cassette subfamily B member 1 (ABCB1) in cancer cells 61. In the study of ovarian cancer, Zhou et al. 62 found that erastin can overcome docetaxel resistance and enhance docetaxel's antitumor efficacy by inhibiting ABCB1. Whether ferroptosis is involved in erastin's enhancement of docetaxel's efficacy in ovarian cancer remains to be determined.

Previous research has demonstrated that glycolysis is up-regulated in numerous types of tumor cells while oxidative phosphorylation (OXPHOS) is down-regulated (OXPHOS) 63. Gentric et al. 64 found that high-OXPHOS metabolism can enhance the sensitivity of chemotherapy for ovarian cancer and improve the prognosis of patients by upregulating *PML* expression and reducing *PGC-1* transcriptional activity to promote oxidative stress (including increased ROS and lipid peroxidation). Increased ROS content and lipid peroxidation are characteristics of ferroptosis 4. Ferroptosis may be involved in the enhanced sensitivity of chemotherapy by high-OXPHOS. Therefore, targeting high-OXPHOS and promoting ferroptosis may be a promising anti-tumor strategy.

Olaparib is a clinical PARP inhibitor that can induce DNA damage in BRCA-mutated ovarian cancer to produce anticancer effects. However, BRCA wild-type ovarian cancer can repair this DNA damage and cause olaparib resistance 65. Hong et al. 47 found that Olaparib can down-regulate SLC7A11 and induce ferroptosis, which resulted in anticancer effects in ovarian cancer cells (including BRCA wild-type and BRCA mutations). Inhibition of SLC7A11 induced ferroptosis significantly sensitized BRCA wild-type ovarian cancer cells to Olaparib.

Wang et al. 66 found that superparamagnetic iron oxide nanoparticles (SPIONs) (nucleic acid and drug carrier) could induce oxidative stress and ferroptosis in ovarian cancer cells and inhibit their proliferation, invasion, and drug resistance.

Chan et al. 67 established that MAP30 (a bioactive protein, isolated from bitter melon seeds) induces ferroptosis and strengthened the anticancer effect of cisplatin *in vitro* (OVCA433, HEY and HEYA8 ovarian cancer cell lines) *and vivo*.

Xuan et al. 42 found that inhibition of SCD1 and FADS2 could induce ferroptosis, reduce cisplatin resistance, and enhance cisplatin's anti-cancer effect; therefore, inhibition of SCD1 and FADS2 in combination with cisplatin was an effective treatment.

In conclusion, ferroptosis can enhance chemosensitivity and plays a crucial function in ovarian cancer chemotherapy. However, the genetic and metabolic factors of ovarian cancer cell sensitivity are still unclear, which limits the application of ferroptosis inducers. A recent study provided evidence that erastin fails to induce ferroptosis in a series of ovarian cancer cell lines 68. Interestingly, they found that the combination of erastin with ferlixit (a regularly used iron compound to treat anemia) can overcome ferroptosis resistance in ovarian cancer cell lines 68. This might be a way to overcome erastin-induced ferroptosis resistance.

### Immunotherapy and ferroptosis

T cells, a crucial component of the adaptive immune system, can selectively eliminate pathogens and tumor cells and play an important role in cancer immunotherapy 69. Immunotherapy can boost anticancer effects by restoring and enhancing CD8+ T cell activity 70. Previous studies have revealed that immunotherapy can promote ferroptosis in ovarian cancer cells. Wang et al. 8 found that immunotherapy-activated CD8+ T cells can enhance the antitumor efficacy of immunotherapy by inducing ferroptosis in ovarian cancer cells. Mechanistically, activated CD8+ T cells could release interferon-gamma (IFN-γ), which inhibited cystine uptake by downregulating the expression of system Xc-, as a result, promoting lipid peroxidation accumulation and ferroptosis in ovarian cancer cells. Cyst(e)inase is an engineered synthetic therapeutic enzyme that can induce ferroptosis by degrading cysteine and cysteine 71, 72. Wang et al. 8 show that Cyst(e)inase in combination with PD-L1 blockade (an immune checkpoint inhibitor agent) can effectively inhibit the growth of the ID8 ovarian cancer cell line. This combination of drugs promotes lipid peroxidation and ferroptosis to a greater degree than Cyst(e)inase or PD-L1 blocker treatment alone. This might be a way to overcome immunotherapy resistance. In addition, Jiang et al. 73 found that Fe3O4-SAS@PLT (a biomimetic magnetic nanoparticle) can induce ferroptosis in the metastatic tumor, which significantly enhanced the immune efficacy of PD-1 inhibitors. Therefore, immunotherapy-induced ferroptosis in tumor cells is a unique anticancer mechanism that is anticipated to become a new form of cancer treatment.

### Radiotherapy and ferroptosis

In the past, radiotherapy was often utilized to treat individuals with ovarian cancer. It was the backbone of adjuvant therapy for many years, but it was supplanted by cisplatin about three decades ago. Nevertheless, it remains a beneficial therapy for individuals with recurrent and refractory illnesses since it can result in a longer DFS 74. Radiotherapy can induce oxidative damage and lipid peroxidation in all cellular compartments 75. The accumulation of toxic lipid peroxidation is related to ferroptosis 4. However, there are few studies on the association between radiotherapy and ferroptosis. Lang et al. 7 revealed that radiotherapy can induce ferroptosis in ID8 ovarian cancer cells. Mechanistically, Radiotherapy suppressed the expression of SLC7A11 by activating the* ataxia telangiectasia mutant gene (ATM)*, ultimately resulting in the accumulation of lipid peroxidation and ferroptosis in ovarian cancer cells. In order to explore whether inducing ferroptosis can enhance the effect of radiotherapy, they pretreated ID8 cells with ferroptosis inducers (such as erastin, FINs, etc.) and then irradiated these cells. The results showed that inducing ferroptosis can reduce cell survival compared to radiotherapy alone in ID8 cells. This indicates that inducing ferroptosis can enhance the effect of radiotherapy. The use of targeted ferroptosis and radiotherapy together could become a new way to treat cancer.

## Future Perspective and Conclusion

Ferroptosis is a novel kind of regulated cell death distinct from autophagy, apoptosis, and necrosis. The precise mechanism of ferroptosis is still unknown. In its research, accumulating evidence shows that it involves many signal pathways and is closely related to the genesis, development, and metastasis of tumors. Its importance in tumor treatment has attracted extensive attention.

**Figure 1:**
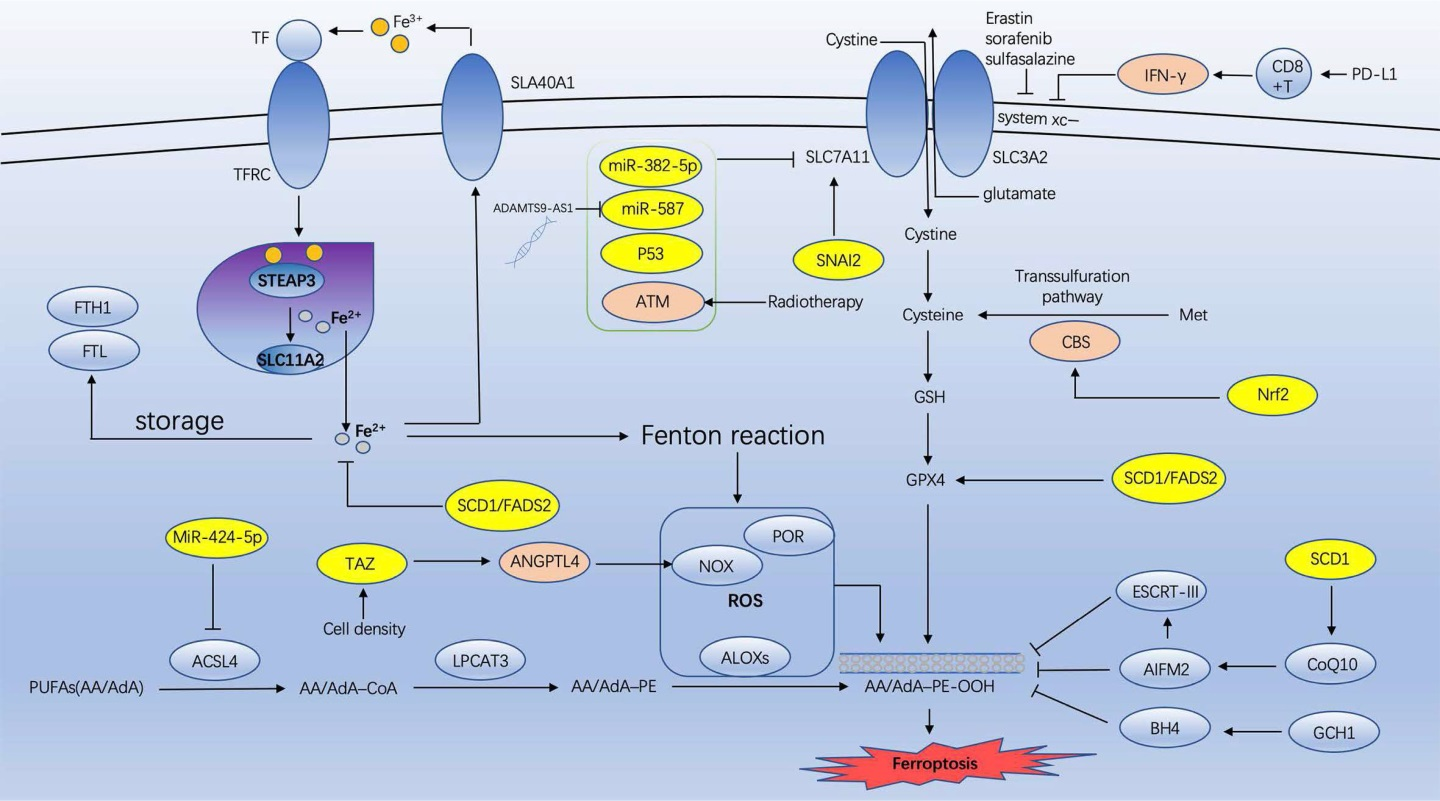
** The possible mechanism of targeting ferroptosis enhancing anti-ovarian cancer** (IFN-γ: interferon-gamma; CBS: cystathionine-β-synthase; Nrf2: erythroid 2-related factor 2; SCD1: sterol CoA desaturase 1; GSH: glutathione; GPX4: glutathione peroxidase 4; ATM: Ataxia- Telangiectasia mutated gene; ACSL4: acyl-CoA synthetase long chain family member 4; LPCAT3: lysophosphatidylcholine acyltransferase 3; NOXs: NADPH oxidases; TAZ: transcriptional coactivator with PDZ-binding motif).

Ovarian cancer is among the most prevalent malignant tumors in females. It has the greatest mortality rate of all gynecological malignancies. Since Dixon proposed ferroptosis in 2012 4, numerous researchers have investigated its effects on various tumors, including ovarian cancer. However, the majority of research has been conducted on ovarian cancer cell lines, for instance, how ferroptosis inhibits the development of ovarian cancer cells. This article reviews the recent research on the association between ovarian cancer and ferroptosis, including the possible mechanism of targeting ferroptosis enhancing anti-ovarian cancer (Figure 1) and the mechanism of ferroptosis in various ovarian cancer cell lines (Table 1).

Presently, an increasing number of studies have demonstrated that ferroptosis can inhibit the progression of ovarian cancer cells, and ferroptosis in combination with chemotherapy, radiotherapy, and immunotherapy can enhance the tumor-inhibiting effect. In the future, targeting ferroptosis may become a novel treatment for ovarian cancer.

## Figures and Tables

**Table 1:** The mechanism of ferroptosis in various ovarian cancer cell lines.

Author year	Ovarian cancer cell line	Mechanism	Refs
Ma et al., 2021	HO8910/SKOV3	MiR-424-5p abrogates ferroptosis in ovarian cancer through targeting ACSL4	28
Cai et al., 2022	OVCAR3/CAOV-3	Long non-coding RNA ADAMTS9-AS1 attenuates ferroptosis by targeting microRNA-587/SLC7A11	29
Sun et al., 2021	SKOV-3	Lidocaine promoted ferroptosis by targeting miR-382-5p /SLC7A11 axis in Ovarian cancer	34
Yang et al., 2020	CAOV2	A TAZ-ANGPTL4-NOX2 axis regulates ferroptosis and chemoresistance in EOC	36
Yang et al., 2021	CAOV2	The Hippo pathway effector YAP promotes ferroptosis via the E3 ligase SKP2	38
Tesfay et al., 2019	OVCAR-4/COV362	SCD1 protects ovarian cancer cells from ferroptosis	40
Xuan et al., 2022	OVCA433	SCD1/FADS2 fatty acid desaturases equipoise lipid metabolic activity and redox-driven ferroptosis in ascites-derived ovarian cancer cells	42
Hong et al., 2021	HEY/A2780/SKOV3	PARP inhibition promotes ferroptosis via suppressing SLC7A11 by P53 in ovarian cancer	47
Jin et al., 2022	SKOV3/A2780/CAOV3	SNAI2 promotes the development of ovarian cancer through regulating ferroptosis	51
Liu et al., 2020	SKOV3/OVCA429	Activation of the reverse transsulfuration pathway through NRF2/CBS confers erastin-induced ferroptosis resistance	54

## Abbreviations

OC: ovarian cancer
OS: overall survival
TF: transferrin
TFRC: transferrin receptor
FTL: ferritin light chain
FTH1: ferritin heavy chain 1
ROS: reactive oxygen species
PUFAs: polyunsaturated fatty acids
AA: arachidonic acid
AdA: adrenic acid
ACSL4: acyl-CoA synthetase long chain family member 4
LPCAT3: lysophosphatidylcholine acyltransferase 3
NOXs: NADPH oxidases
GSH: Glutathione
GPX4: glutathione peroxidase 4
YAP: yes-associated protein 1
TAZ: transcriptional coactivator with PDZ-binding motif
SKP2: S-phase kinase-associated protein 2
SCD1: Sterol CoA desaturase 1
Nrf2: nuclear factor erythroid 2-related factor 2
CBS: Cystathionine-β-synthase
FZD7: Wnt receptor Frizzled 7
ABCB1: ATP binding cassette subfamily B member 1
IFN-γ: interferon-gamma
ATM: Ataxia- Telangiectasia mutated gene
